# Duplication of *C7orf58*, *WNT16* and *FAM3C* in an Obese Female with a t(7;22)(q32.1;q11.2) Chromosomal Translocation and Clinical Features Resembling Coffin-Siris Syndrome

**DOI:** 10.1371/journal.pone.0052353

**Published:** 2012-12-27

**Authors:** Jun Zhu, Jun Qiu, Gregg Magrane, Malak Abedalthagafi, Andrea Zanko, Mahin Golabi, Farid F. Chehab

**Affiliations:** 1 Department of Laboratory Medicine, University of California San Francisco, San Francisco, California, United States of America; 2 Department of Pathology, University of California San Francisco, San Francisco, California, United States of America; 3 Department of Pediatrics, University of California San Francisco, San Francisco, California, United States of America; University of Kentucky, United States of America

## Abstract

We characterized the t(7;22)(q32;q11.2) chromosomal translocation in an obese female with coarse features, short stature, developmental delay and a hypoplastic fifth digit. While these clinical features suggest Coffin-Siris Syndrome (CSS), we excluded a CSS diagnosis by exome sequencing based on the absence of deleterious mutations in six chromatin-remodeling genes recently shown to cause CSS. Thus, molecular characterization of her translocation could delineate genes that underlie other syndromes resembling CSS. Comparative genomic hybridization microarrays revealed on chromosome 7 the duplication of a 434,682 bp region that included the tail end of an uncharacterized gene termed *C7orf58* (also called *CPED1*) and spanned the entire *WNT16* and *FAM3C* genes. Because the translocation breakpoint on chromosome 22 did not disrupt any apparent gene, her disorder was deemed to result from the rearrangement on chromosome 7. Mapping of yeast and bacterial artificial chromosome clones by fluorescent in situ hybridization on chromosome spreads from this patient showed that the duplicated region and all three genes within it were located on both derivative chromosomes 7 and 22. Furthermore, DNA sequencing of exons and splice junctional regions from *C7orf58*, *WNT16* and *FAM3C* revealed the presence of potential splice site and promoter mutations, thereby augmenting the detrimental effect of the duplicated genes. Hence, dysregulation and/or disruptions of *C7orf58*, *WNT16* and *FAM3C* underlie the phenotype of this patient, serve as candidate genes for other individuals with similar clinical features and could provide insights into the physiological role of the novel gene *C7orf58*.

## Introduction

The uncovering of genes that cause clinically severe genetic disorders often reveals a critical physiological role for these genes. Recently, mutations that cause Coffin-Siris syndrome (CSS) (MIM 135900) [1], [2], a rare congenital disorder, were unveiled and occurred in chromatin remodeling genes of the SWI/SNF complex [3], [4], [5]. Other congenital anomalies that overlap with clinical features of CSS, such as the brachymorphism-onychodysplasia-dysphalangism syndrome [6], [7], [8], Cornelia de Lange syndrome [9], [10], Mabry syndrome, Nicolaides–Baraitser syndrome (NCBRS), DOOR syndrome (deafness, onychodystrophy, osteoodystrophy, mental retardation, fetal alcohol syndrome, fetal hydantoin/phenytoin embryopathy and trisomy 9 [5], complicate the differential diagnosis of these disorders. To help in their differential diagnosis, clinical algorithms were proposed [5], [11] without however, any molecular basis. To begin the identification of genes that share clinical features with CSS and related disorders, we characterized the translocation breakpoints in a female with a *de novo* t(7;22)(q32;q11.2) chromosomal translocation [12], who presented with clinical features that resemble CSS [11]. We found that a duplication encompassing the *WNT16* and *FAM3C* genes and the tail end of a novel gene, termed *C7orf58*, underlie the clinical phenotype of this patient. The identification of mutations in these genes among similarly affected patients could provide insights into their biological roles and may help resolve their ambiguous diagnoses.

## Methods

### Ethics Statement, DNA Samples and Leptin Radioimmunoassay

Blood samples were obtained from the index patient, following informed written consent and approval by an institutional review board (IRB) of the UCSF Committee on Human Research (CHR). Leftover de-identified DNA specimens from Caucasian individuals were obtained from the UCSF Molecular Diagnostics Laboratory. Such decoded biological specimens do no meet the definition of human subject research as specified by the U.S. Department of Health and Human Services regulations 45 CFR 46.101(b).4 and are therefore not considered human subjects. Thus, IRB approval and informed consent are neither applicable nor necessary.

Circulating leptin levels from the patient were determined in duplicates from plasma using a radioimmunoassay from Linco Research (Millipore). DNA extractions were performed by a standard proteinase K method.

### Exome Sequencing and Bioinformatics Analysis

Next Generation Sequencing (NGS) was performed on 3 µg of peripheral blood DNA using an Illumina HiSeq following library preparation and exome enrichment with Agilent SureSelect Human All Exon v4 51 Mb kit. Paired-end readings resulted in 50.39 million reads and 7.56 GigaBases, representing an overall average coverage of 100×. The raw sequences were aligned to the human genome build hg19 using Burrows-Wheeler Aligner (BWA) software and the resulting SAM files analyzed for single nucleotide polymorphisms (SNPs) and insertions/deletions (INDEL) with PICARD, SAMTOOLS and GATK. SNPs and INDELs were further analyzed and annotated with ANNOVAR.

### Array Based Comparative Genomic Hybridization

Array CGH microarray was performed at Baylor College of Medicine. Briefly, normal and patient DNA samples were labeled with Cy3 and Cy5 fluorescent dyes and hybridized to a 60-mer oligonucleotide human genome microarray platform (244K Agilent, Santa Clara, CA) at a probe density of 5–8 kb. The results were analyzed with the Agilent DNA Analytics software. Copy number variations were mapped onto the human genome database hg18 and the variants, when applicable, identified in the Toronto Database of Genomic Variation (DGV).

### Contig Mapping and Fluorescent In Situ Hybridization

Yeast and bacterial artificial chromosome clones were obtained from the Human Genome Center at the National Institutes of Health (Eric Green’s chromosome 7 library) and from Genome Systems Inc. (St. Louis MI). Plasmid DNA was extracted by standard methods and a one-microgram aliquot labeled by nick translation using FITC-dUTP. Metaphase cells were enriched in synchronized peripheral blood cultures according to the method of Yunis [13], fixed in 3∶1 methanol:acetic acid and dropped on glass slides. Labeled DNA probes were hybridized to chromosome spreads from this patient as previously described [14]. Additional FISH probes consisted of gel purified Bam HI and Hind III restriction fragments derived from BAC 146J04, consisting of 11,085 bp, 27,092 bp and 17,923 bp respectively. At least two spreads from each hybridization were fully karyotyped and analyzed.

### Sanger Dideoxy DNA Sequencing and Analysis

PCR primers (Table S1) bracketing the exon-intron boundaries of *WNT16*, *FAM3C* and *C7orf58* were used to amplify genomic DNA. The targeted regions covered all exons from these three genes, including at least 100 bp flanking the splice donor and acceptor sites of each exon. In addition, the DNA sequence of the upstream 2745 bp from the initiation codon of *C7orf58* was determined using six promoter specific PCR amplicons. Amplification reactions were set up using a 2X master mix (Affymetrix) and processed for 35 cycles at 95°C, 55°C, 72°C for 30 seconds each. The PCR amplicons were purified from residual PCR primers and their DNA sequence determined in both orientations by Sanger dideoxy sequencing using either PCR primer. The reaction products were then fractionated and analyzed on an ABI 3730 XL DNA sequencer. DNA sequences generated by the amplicons were analyzed and compared to their respective reference Genbank sequences using a DNA sequence Aligner obtained from CodonCode Corporation (Dedham, MA).

### Determination of Allele Frequencies in SNPs

Allele frequencies were determined using 20–40 anonymous DNA samples from Caucasian individuals, who requested cystic fibrosis carrier screening at the UCSF Molecular Diagnostics Laboratory. The SNPs and number of individuals tested were in *C7orf58*: rs78458119 (n = 20), rs33990520 (n = 25), rs68173311 (n = 23), INDEL GAAAA (n = 21), in *WNT16*: rs75556099 (n = 40), rs3832519 (n = 32), and in *FAM3C*: INDEL AAAACTT (n = 20), rs3837124 (n = 20).

### Bioinformatics Mutation Predictions

Analysis of the transcription factor binding sites in the putative promoter region was determined by the PROMO [15] and TESS [16] tools. Analysis of missense mutations was performed using the bioinformatic tools SIFT [17], PolyPhen2 [18] and Mutation Taster [19]. Putative mutations affecting splice donor, splice acceptor and branch point sites were analyzed with Human Splicing Finder [20] and Spliceport [21]. Phosphorylation sites were predicted by NetPhos [22]. Micro RNA binding sites were queried for 15 nucleotides on either side of the SNP against the mature miRNAs database using the miRBase search engine [23] set at maximum E-value of 10 and using the BLASTN search method.

## Results

### Clinical Synopsis

The clinical findings of the index female patient reported in this study were initially reported in [12]. She is a Caucasian girl with a 46, XX karyotype and a *de novo* apparently balanced chromosome translocation, 46, XX, t(7;22)(q32.1;q11.2). Her parents had normal karyotypes. She attended a special educational resources classroom throughout her schooling. A developmental assessment at 9 years chronological age showed a performance below 6.5-year level and a delay of about three years. Her strengths were in verbal reasoning and her weaknesses in concentration, visual-motor pursuits, fine motor development and poor gross motor with unsteady gait. In earlier childhood, she experienced frequent bouts of respiratory infections and otitis media, but did not require hospitalization. At 9 years, it was noted that her hair was of normal consistency and growth pattern. She had experienced two grand mal seizures with normal EEG and normal MRI. She had two congenital melanocytic nevi surgically excised, one from the right posterior scalp and the other from the left clavicle. An ulcerated pyogenic granuloma was excised from the left mid thoracic spine. At 11 years of age, she was in 4^th^ grade with special educational resources and had exhibited considerable weight gain and a decline in gross motor abilities. At that time, her height was 129.5 cm (5–10%), weight 57.7 Kg (>97%) head circumference 54.25 cm (75%). Her features and clinical history were not felt to be consistent with Prader-Willi Syndrome (PWS) and FISH/methylation studies for PWS were normal. She had a BMI of 34.4 and her leptin levels were 31.5 ng/ml. Endocrine evaluations found no etiology for her clinical presentation and her hearing was within normal limits. Her vision was 20/60 with corrective glasses prescribed. While the primary concern remained her obesity, she had no self-restraint and food intake was to be continuously monitored. She also manifested some obsessive-compulsive behaviors. Review of multiple photographs at 9, 10 and 11 years of age likely excluded a CSS diagnosis based on atypical features of facial coarseness, sparse scalp hair, excessive amount of hair in eyebrow/eyelash, dysplasia and aplastic nails (Bryan Hall, personal communication). At 22 years old, she weighed 88.9 Kg, measured 137.2 cm and had a BMI of 47.2. She only gained 7.6 cm in height over the past 10 years. Hyperphagia, obesity and reduced cognition remain the predominant issues.

### Exome Sequencing

There were no deleterious mutations such as stops or frameshift mutations in any of the chromatin remodeling genes SMARCA2, SMARCA4, SMARCE1, SMARCB1, ARID1A and ARID1B, recently reported to be mutated in CSS [3], [4]. However, synonymous and non-synonymous single nucleotide variations were found in SMARCA2, SMARCA4, SMARCB1 and ARID1B and in other members of the SMARCA and ARID gene families, SMARCA5, SMARCAD1, ARID3A, ARID3C and ARID4A (Table 1). All these variants had common minor allele frequencies (MAF), which preclude them from underlying this patient’s rare phenotype. Furthermore, no deleterious mutations were found in genes known to cause morbid obesity such as leptin, leptin receptor, melanocortin 4 receptor, POMC, adiponectin and adiponectin receptor.

**Table 1:** SNPs revealed by exome sequencing in chromatin remodeling genes of the index patient.

Gene	Nucleotide	Protein	dbSNP	Genotype	SNV	MAF
**SMARCA2**	c.G3672A	p.E1224E	rs6601	0/1	syn	0.203
**SMARCA4**	c.T1524C	p.H508H	rs7935	0/1	syn	0.312
**SMARCA4**	c.T4887C	p.D1629D	rs7275	0/1	syn	0.22
SMARCA5	c.T2424C	p.N808N	rs13139128	1/1	syn	0.996
SMARCAD1	c.C1839T	p.D613D	rs6823404	0/1	syn	0.432
SMARCAD1	c.T902C	p.V301A	rs7439869	0/1	nonsyn	0.287
**SMARCB1**	c.C707G	p.P236R	rs75919464	1/1	nonsyn	0.228
**ARID1B**	c.G1959A	p.A653A	rs3734441	1/1	syn	0.499
ARID3A	c.A225G	p.P75P	rs1799595	1/1	syn	0.115
ARID3A	c.G150A	p.E50E	rs3826948	0/1	syn	0.496
ARID3A	c.T1161C	p.N387N	rs12608658	1/1	syn	0.056
ARID3A	c.C1320T	p.A440A	rs6510986	1/1	syn	0.295
ARID3A	c.A1650G	p.G550G	rs1051504	1/1	syn	0.374
ARID3A	c.G1666A	p.G556S	rs1051505	1/1	nonsyn	0.403
ARID3C	c.G72A	p.P24P	rs13283357	0/1	syn	0.234
ARID3C	c.T1003G	p.C335G	rs3808869	0/1	nonsyn	0.435
ARID3C	c.A1061G	p.D354G	N/A	0/1	nonsyn	N/A
ARID4A	c.A2171G	p.N724S	rs2230098	0/1	nonsyn	0.035
ARID4A	c.A2335G	p.T779A	rs1051858	0/1	nonsyn	0.384

Bolded are the genes mutated in Coffin- Siris Syndrome. Standard nomenclatures for cDNA and protein variations (hg19) with their dbSNP reference numbers are listed. Genotypes variations from the reference hg19 genome sequence are indicated as 0/1 and 1/1 for heterozygous and homozygous variants, respectively. The effect of each SNP, denoted as single nucleotide variation (SNV), is shown as either synonymous (syn) or non-synonymous (nonsyn). Minor allele frequencies (MAF) are taken from the 1000 Genomes Project. N/A denotes non-available information.

### Characterization of the Chromosomal Translocation by Array CGH

**Figure 1:**
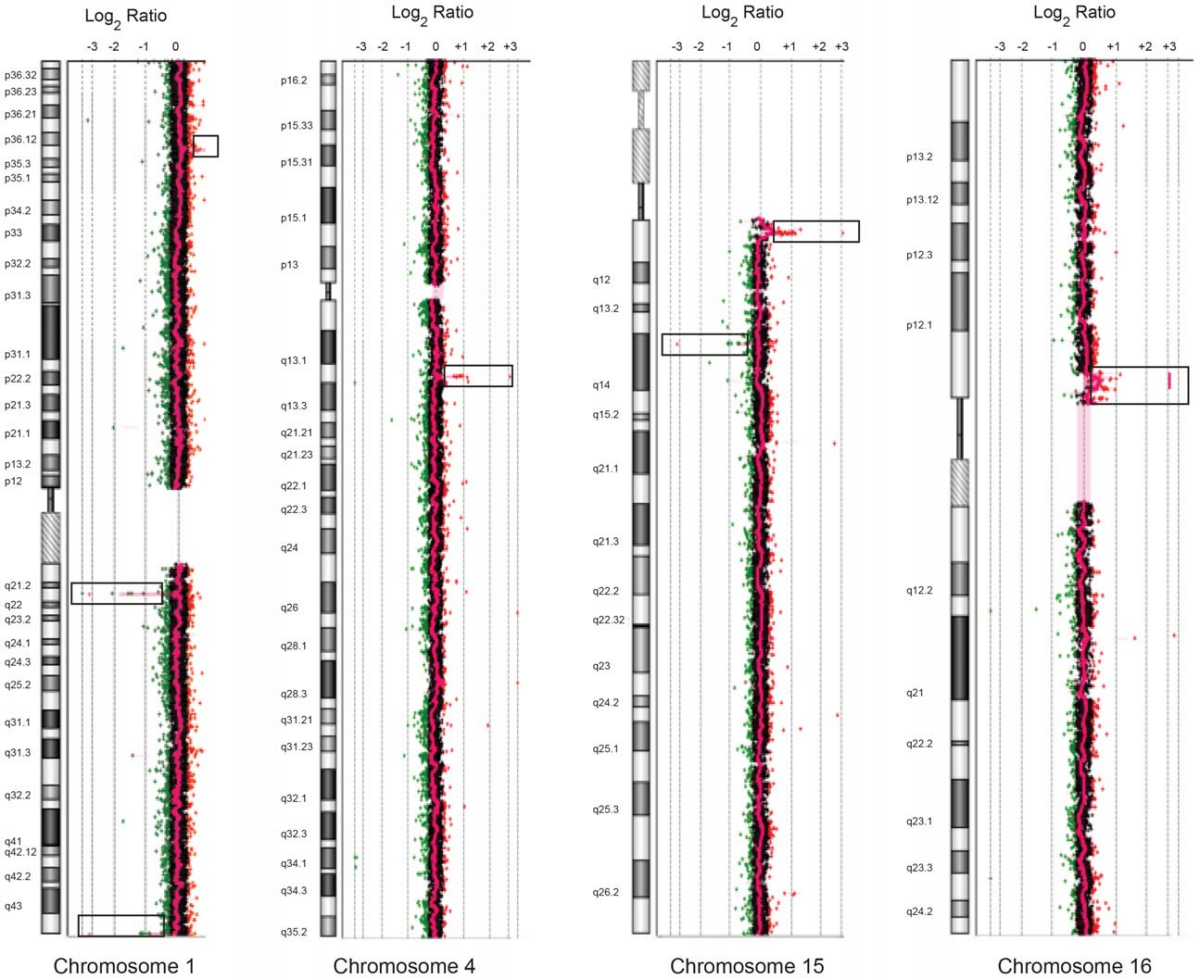
CNVs on chromosomes 1, 4, 15 and 16 of the index patient revealed by Agilent 244K human genome CGH microarray hybridization. The profiles shown represent the averaged combined hybridization data of patient vs. reference DNA. Regions of gain and losses are boxed and depicted as positive or negative log_2_ ratios relative to the midline that denotes no gain/no loss of DNA sequences.

**Figure 2:**
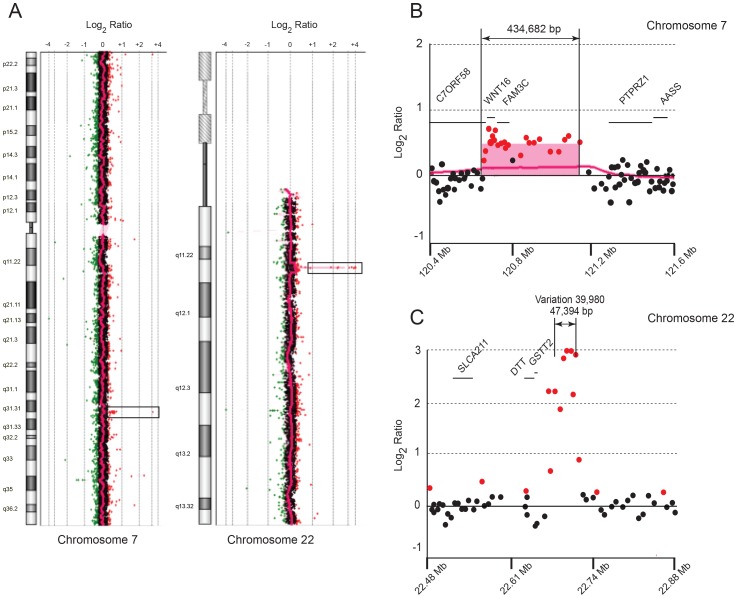
Array CGH array of the index patient showing duplication and amplification of DNA sequences on chromosomes 7 and 22. (**A**) Chromosome 7 and 22 plots displaying gains of DNA sequences, shown in boxes, in the 7q31.3 and 7q22 regions. (**B, C**) Detailed plots of oligonucleotide probes from the microarray along each region of interest denoting the genes and their chromosomal coordinates (hg18) in the duplicated and amplified regions of chromosomes 7 and 22, respectively.

Array CGH analysis of this patient’s DNA revealed copy number variations (CNVs) on chromosomes 1, 4, 15 and 16, shown in Figure 1 and annotated in Table 2. On chromosome 7, a unique duplication of 434,682 bp was revealed and defined by hg18 (Human Genome Build 36.3) between coordinates 120,724,175–121,158,857 (Fig. 2A) and encompassed the *WNT16*, *FAM3C* and part of *C7orf58*. One boundary of the duplication was located in intron 22 of *C7orf58*, while the other was approximately 44 Kb upstream of the protein tyrosine phosphatase gene (*PTPRZ1*). This duplication was not previously reported and occurs at 7q31.31.

**Table 2:** Copy number variation (CNV) identified by array CGH in the patient.

Chr.	Genomic position hg18	Variation No.	Gain (bp)	Loss (bp)	Frequency (n)
1	25,482,036–25,536,138	38960	54,102	–	Watson
1	150,823,073–150,862,088	38961	–	29,382	Watson
1	246,795,223–246,862,088	66541	–	66,865	182/450
4	69,057,735–69,165,872	38966	108,137	–	Watson
15	19,805,960–20,220,475	2182	414,515	–	81/269
15	32,517,513–32,594,948	1960	–	77,435	48/50
16	32,105,104–33,539,082	38978	1,433,978	–	28/43
22	22,677,959–22,725,353	39,980	47,394	–	22/39, 24/40

Shown are the CNV coordinates in hg18 (Build 36.3) for the gains and losses of DNA sequences on the listed chromosomes (Chr.) The number of individuals or occurrence in the Watson genome are indicated in the frequency data, which are derived from the Toronto Database of Genomic Variants (DGV).

On chromosome 22, a 47,394 bp region was amplified at multiple copies (>2) around the translocation breakpoint region on chromosome 22q11.2 (Fig. 2B) but was previously reported as variation 39,980 in the DGV database. This CNV maps between coordinates 22,677,959 and 22,725,353 in hg18. It was also reported in the Watson genome [24] and in 24 of 30 [25] and 22 of 39 individuals in the HapMap study [26]. Therefore, based on its common occurrence among normal individuals, this amplification of DNA sequences on chromosome 22q11.2 is unlikely to contribute to the phenotype of this patient. Therefore, the duplication on chromosome 7 represents her most significant molecular lesion.

### FISH Mapping of the Translocation Breakpoint and the Duplicated Region Encompassing *C7orf58, WNT16* and *FAM3C*

**Figure 3:**
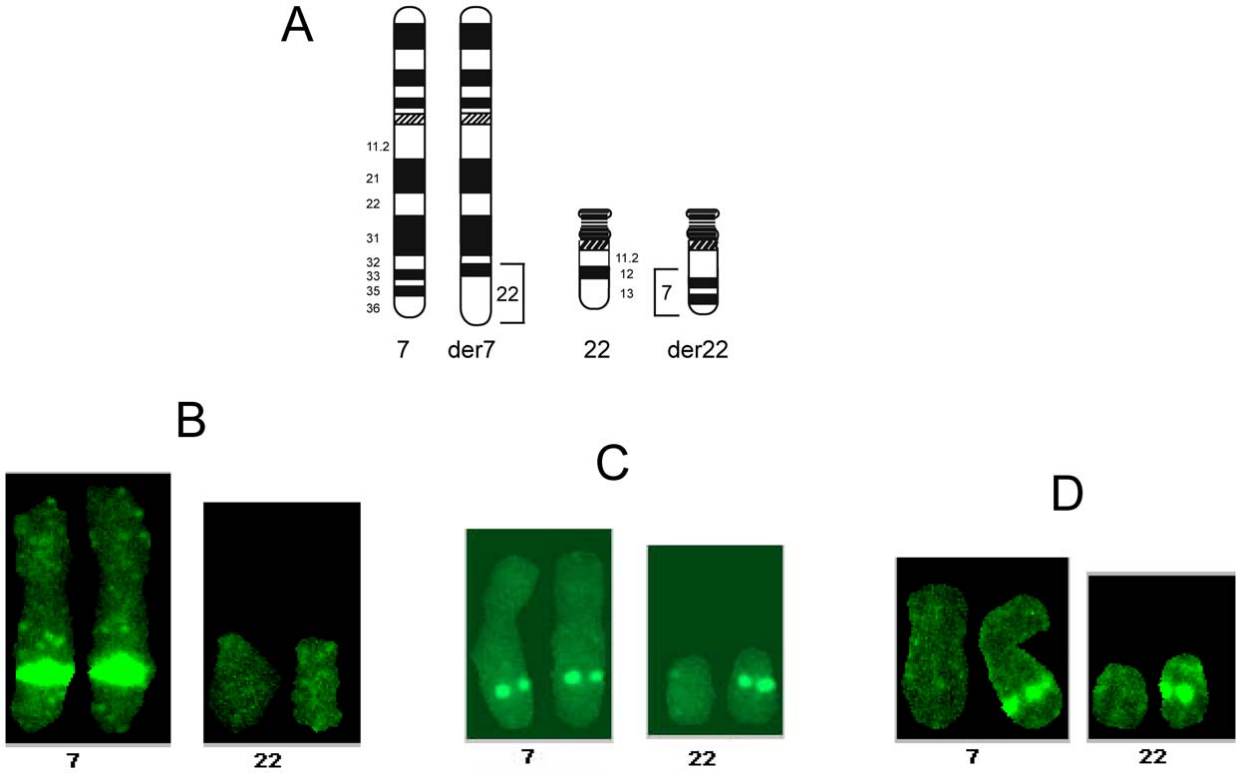
Representative fluorescent *in situ* hybridization (FISH) patterns of DNA probes onto chromosomes 7, 22 der(7) and der(22) from this patient’s chromosome spreads. (**A**) Schematic drawing of normal chromosomes 7 and 22 and derivative chromosomes der(7) and der(22), showing the translocated chromosomal regions. The three panels below depict each the typical hybridization of a probe located (**B**) on chromosomes 7, der(7), (**C**) on chromosomes 7, der(7), der(22) and (**D**) chromosomes 7 and der(22).

**Figure 4:**
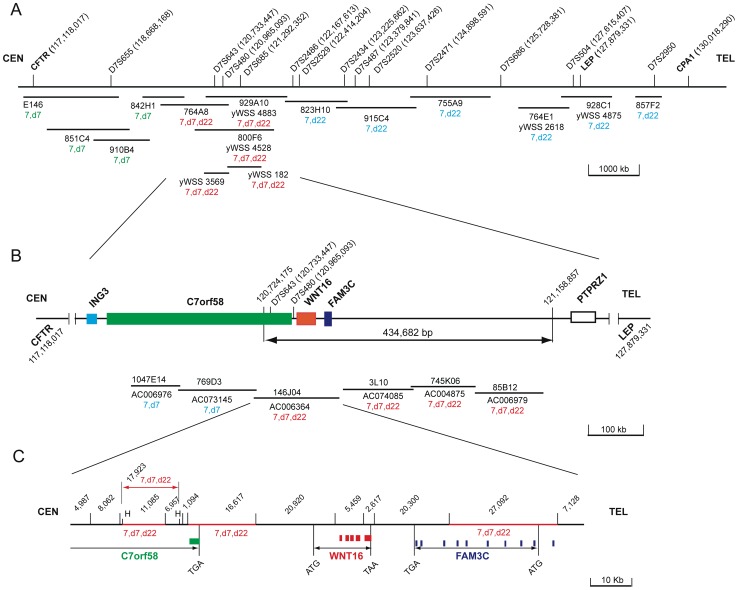
FISH maps of YAC and BAC DNA clones from chromosome 7q31.1 in and around the duplicated region. The hg19 genomic coordinates of each marker and gene are indicated next to its designated name. The centromeric (CEN) and telomeric (TEL) directions are specified for orientation on the chromosome. Clones are denoted by their clone names, plate coordinates or/and Genbank designations. Each clone or restriction fragment is also denoted by its hybridization onto the patient’s chromosomes 7, der(7) and/or der(22). (**A**) FISH map of a 12.9 Mb region of chromosome 7 extending from the CFTR to the CPA1 genes. The denoted YAC probes represent three distinct areas of hybridization relative to the chromosome 7 translocation breakpoint, namely distal (7, der22), at or around the breakpoint (7, der7, der22) and proximal to the breakpoint (7, der7). (**B**) FISH map of BAC clones at and proximal to the 434,682 bp duplicated region, spanning the tail end of *C7orf58* and the entire *WNT16* and *FAM3C* genes. Note that all BAC clones within the duplicated region map to 7,d7,d22. (**C**) Bam HI restriction map, except for two designated Hind III (H) sites, of BAC 146J04 showing the FISH mapping of Bam HI and Hind III restriction fragments, indicating hybridization to 7,der(7) and der(22). The sizes in bp of each restriction fragment are shown and the exons in each gene displayed with solid boxes. The initiation (ATG) and termination (TGA, TAA) codons denote the gene boundaries except for C7orf58, which is only spanned by its last exon and part of the adjacent intron.

Various yeast and bacterial artificial clones from chromosome 7 were mapped by fluorescent *in situ* hybridization (FISH) onto chromosome spreads from this patient. The aim of such a mapping was to sublocalize the duplicated region by determining whether a specific DNA clone hybridizes onto her chromosomes 7 and der(7), 7 and der(22) or 7, der(7) and der(22). A representative FISH from each pattern is shown in Figure 3B–D. Towards these ends, we derived YAC clones from a 12.9 Mb chromosomal map region [27] extending from 7q31.2–7q32.1 and defined by the cystic fibrosis transmembrane regulator (CFTR) and carboxypeptidase 1 (CPA1) genes. Figure 4A displays a map of three regions depicted each by a FISH hybridization pattern. One region displayed hybridization to six YACs (857F2, yWSS4875, yWSS2618, 755A9, 915C4, 823H10) onto chromosomes 7 and der (22), indicating that their corresponding DNA sequences from chromosome 7q were translocated to der(22). Another region characterized by its hybridization patterns on chromosomes 7 and der (7) included 4 YACs (E146, 851C4, 910B4, 824H1) and represented DNA sequences that were retained on der (7). A third region that lied between the two previous regions was spanned by five YACs (764A8, yWSS4883, yWSS4528, yWSS3569, yWSS182) and displayed hybridization to chromosomes 7, der(7) and der(22), suggesting that these clones span the translocation breakpoint on chromosome 7.

To refine the FISH map within the region of interest that showed hybridizations to chromosomes 7, der(7) and der(22), we mapped BAC clones within and around the duplicated region (Fig. 4B). Two clones (1047E14, 769P3) located within the last intron of *C7orf58* and outside of the duplicated region hybridized to chromosomes 7 and der(7). However, all other clones located in the duplicated region hybridized to chromosomes 7, der(7) and der(22), showing that the duplication extends to the segment of chromosome 7 that has been translocated to der(22) and was thus not tandem duplicated on der(7). Incidentally, BAC 146J04 spans the tail end of *C7orf58* and all of *WNT16* and *FAM3C*. Therefore, we mapped restriction fragments DNA probes from BAC 146J04 to confirm that those genes had indeed been translocated onto der(22). The map in Figure 4C shows that the 11,085 bp and 16,617 bp Bam HI fragments, the 17,923 bp, Hind III fragment and the 27,092 bp Bam HI fragment from the opposite end, all map to chromosome 7, der(7) and der(22), thus corroborating their location on der(7) and translocation to der(22).

### C7orf58, WNT16 and FAM3C

The three genes identified within the duplicated region of chromosome 7 are *C7orf58* (also called *FLJ21986* and *CPED1*), *WNT16* and *FAM3C* (also called *GS3786*). The closest two genes on either side of the duplicated region are *ING3,* 13 kb upstream of *C7orf58*, and *PTPRZ1*, 475 kb downstream of *FAM3C*. Information about the expression and structure of *C7orf58 WNT16,* and *FAM3C* was derived from NCBI gene resources (http://www.ncbi.nlm.nih.gov/gene). The novel *C7orf58* gene spans approximately 306,038 bp from the initiation to termination codons of its longest isoform and generates at least three protein isoforms of 117.6 kD, 89.2 kD and 22.6 kD that are encoded by exons 1–23 (excluding exon 18), 1–18 and 1–3A, respectively. Another 64.6 kD isoform, which originates from an alternate promoter is encoded by exons 5–18. The *WNT16* gene spans approximately 16 kb from the initiation to termination codons. It encodes two protein isoforms of 40.5 kD, 40.7 kD, based on an alternate initiation site of translation that encompasses exon 1A or exon 1B, The two resulting isoforms, termed *WNT16*a and *WNT16*b differ by 30 amino acids at their amino terminus. *FAM3C*, a member of a gene family with sequence similarity, encodes a ubiquitously expressed 227 amino acid secretory cytokine-like protein [28]. It spans approximately 32.5 kb and encodes a polypeptide chain of 24.7 kD, encompassing 9 exons. All three genes are expressed in multiple tissues except for the *WNT16*a isoform, which is restricted to the pancreas [29].

### Mutation Analysis of *C7orf58*, *WNT16* and *FAM3C*

We found 21 SNPs in *C7orf58*, 5 SNPs in *WNT16* and 4 SNPs in *FAM3C* (Table S1). Two previously unreported SNPs, INDEL GAAAA and INDEL AAAACTT were located in intron 13 of *C7orf58* and intron 7 of *FAM3C*, respectively. Allelic frequencies of 8 SNPs (rs78458119, rs3390520, rs68173311, INDEL GAAAA, rs75556099, rs3832519, INDEL AAAACTT, rs3837124) occurred at fairly common frequencies (range 0.065–0.5) to justify their clinical significance as the sole cause of a rare disorder. However, their individual or combined effects may add to the effect of the duplication. Table S1 also lists the potential effects of SNPs that were analyzed with various online bioinformatics tools. Overall, among the 30 SNPs, there were 13 SNPs with no predicted effects, 6 and 2 SNPs with putative splice acceptor and splice donor defects, 4 SNPs with potential miRNA binding sites, 1 SNP with a predicted transcription factor binding site, 2 SNPs with amino acid substitutions (A551G, D774N), both representing a conservative change, 1 non-synonymous SNP (T253I) that would lose a putative threonine phosphorylation site, 1 non-synonymous SNP with a deleterious amino acid change (G72R), and a protein truncating mutation (CCCA INDEL) in the *WNT16*A isoform.

In *C7orf58*, there were 11 potentially functional SNPs (pfSNPs). The most significant change was rs2110277 in the alternate promoter region of the 64.6 kD isoform, which is predicted to differentially bind transcription factors (Fig. S1). The A allele constitutes a potential binding site for the glucocorticoid receptor beta (GR-β), the yin-yang (YY1) activator/repressor and the CCAAT-enhancer-binding proteins (CEBPα, CEBPβ), while the G allele eliminates these DNA binding sites and generates instead a DNA sequence recognized by the early growth response (EGR) transcription factors 1–3. No SNP variation was found in the promoter region upstream of the 117.6 kD isoform. Cryptic acceptor splice sites were likely to be generated by rs55666906, rs798949 and rs68173311 while reduced splice acceptor efficiencies were predicted by rs55666906 and rs111663168. One SNP (rs61128227), located just 7 nucleotides downstream of the intron 12 splice donor site, creates a new splice donor site, which when used, would cause the insertion of three in-frame amino acids, producing an elongated protein. Furthermore, two SNPs (rs41281692, rs10953934) that cause missense mutations (A551G, D554N) were found to constitute conservative changes and are unlikely to be functionally significant. Two other SNPs (rs798948, rs10241888) were predicted to bind mature miRNAs (hsa-miR-449c-5p and hsa-miR-4733-3p) even though rs798948 is a synonymous amino acid change. Previous studies have shown that a synonymous variant could cause a significant change in miRNA binding site [30].

In *WNT16*, the most deleterious effect was the homozygous CCCA DEL allele (rs755560) at codons 6–7, which produces a truncated *WNT16*a polypeptide of 46 amino acids instead of the normal 361 amino acids. Also, the missense mutation G72R (rs2908004) was found to have a deleterious effect (by SIFT and SNPeffect), whereas the T253I (rs2707466) was a tolerant change by SIFT. On the other hand, the threonine residue anticipated to be phosphorylated by NetPhos (score 0.833) would be lost by the substitution to isoleucine and the proband is homozygous for the isoleucine allele. Of the two other SNPs located downstream of the termination codon, the T allele of rs17143305 creates a binding site for miRNA hsa-miR-362-5p for which the proband is heterozygous. In contrast, rs3832519 has a lower probability of binding a miRNA and may thus be insignificant.

In *FAM3C*, the proband is heterozygous for the INS allele of the new AAAACTT INDEL, located 13 bp downstream of the intron 7 splice donor site, which is expected (by Spliceport) to reduce the splicing efficiency of the wild-type splice donor site. Furthermore, rs3837124 INDEL A located in a run of 10 consecutive adenines, 82 bp downstream of the splice donor site of intron 7, was not found to result in any significant splice site abnormalities.

## Discussion

The patient in this study was previously reported as a possible case of CSS [12]. However, based on atypical clinical features, the absence of mutations in six chromatin remodeling genes, recently shown to cause CSS [3], [4], and in other chromatin remodeling genes from the SMARCA and ARID gene families, a CSS diagnosis was excluded. Nevertheless, the characterization of her phenotype will prove useful for the diagnosis of disorders resembling CSS such as the brachymorphism-onychodysplasia-dysphalangism syndrome [6], [7], [8], Cornelia de Lange syndrome [9], [10], Mabry syndrome, Nicolaides–Baraitser syndrome (NCBRS), DOOR syndrome (deafness, onychodystrophy, osteoodystrophy, mental retardation, fetal alcohol syndrome, fetal hydantoin/phenytoin embryopathy and trisomy 9 [5].

The absence of any apparent gene disruptions on chromosome 22, implicates the duplications of *C7orf58*, *WNT16* and *FAM3C* as the cullprit genes involved in this particular phenotype. As a result, three different types of molecular lesions could occur in this patient. The most noticeable deleterious effect arises from the duplication of *WNT16* and *FAM3C*, which could lead to an increase in gene expression, resulting in a gain of function of each encoded protein. Indeed, overexpression of *WNT16* was found to result in constitutive WNT signaling [31], [32] and generally, WNT genes have been implicated in a wide range of genetic and cancer disorders [33]. Furthermore, a genome wide association study of height revealed a significant association of rs7776725 in *FAM3C* with bone mineral density [34], consistent with the short height of this patient. Another deleterious effect could result from the disruption of *C7orf58* at its duplicated breakpoint in the last intron, which would truncate its longest isoform. As the pathogenesis of *C7orf58* remains unknown, it is interesting to note that a patient with mental retardation, anxiety disorder and autistic features was reported to have a complex 7q rearrangement that also appeared to disrupt *C7orf58* [35]. While our patient did not exhibit the major types of symptoms as the patient reported by Dauwerse *et al.* [35], it is unclear what is the phenotypic contribution of a disrupted *C7orf58* gene in the context of a large chromosomal rearrangement or a smaller duplication that encompasses multiple genes. Yet another possibility is a translocation-mediated change in chromatin, which could impact on the expression of the nearby gene, *ING3* that is located just 13 Kb upstream of *C7orf58*. Most interestingly, the protein encoded by *ING3* contains a PHD-finger domain, a common motif involved in chromatin remodeling genes [36] that are increasingly being recognized as target genes for developmental disorders, such as recently in Potocki-Shaffer-syndrome, which is caused by disruption of a BRAF-histone deacetylase gene [37]. While no potentially functional SNPs were found by exome sequencing in *ING3*, chromatin disruption could interfere with the regulated expression of its allele near the duplicated region. Interestingly, the association of allelic loss and reduced expression of *ING3* with head and neck cancers [38] is reminiscent of a similar association in chromatin remodeling genes of the SWI/SNF complex in CSS [3], [4] with tumorigenesis and gastric cancer [39], [40], [41].

It is also possible that functional SNPs could independently and further contribute to the deleterious effect of the duplication. Exonic and junctional intronic SNPs in *C7orf58*, *WNT16* and *FAM3C* suggested potentially deleterious SNPs in these three genes. Among these, the two most significant SNPs were rs2110277 in the alternate promoter region of the 64.6 kD isoform of *C7orf58*, which is predicted to differentially bind transcription factors and the DEL protein truncating mutation (rs75556099) at codon 6 of the *WNT16*A isoform that produces an aberrant polypeptide chain of 46 amino acids. The differential binding of transcription factors could alter the expression of *C7orf58* and cause ectopic expression. As for the truncation of the *WNT16a* isoform, its restricted expression to the pancreas [29] coupled with its allelic frequency of 0.312 makes it more likely to be associated with a frequent disorder such as obesity, rather than the rare CSS-related disorders. In this vein, the relationship of *WNT* proteins to adipose tissue is exemplified by *WNT10a*, which suppresses adipocytes differentiation [42] and inhibits obesity in genetically obese mice [43]. Hence, it is conceivable that wild-type *WNT16*a could play a similar role, whereas its deletion mutant allele (rs75556099) could reverse this effect by promoting adipocytes differentiation and causing obesity. While the early onset obesity and hyperphagia of this patient are not associated with endocrine abnormalities, mutations in leptin and leptin receptor were ruled out based on her elevated leptin levels, which correlated with her BMI of 34.4 [44] and with the observation that heterozygous leptin null mutations result in significantly lower than normal leptin levels [45]. Furthermore, exome sequencing did not reveal a deleterious mutation in any of the known obesity genes, suggesting that, if obesity was related to this translocation/duplication, the culprit region would likely fall on any or a combination of genes involved in the duplication.

Overall, the molecular basis of disease in this patient appears to be confined to the three-gene cluster, *C7orf58*, *WNT16* and *FAM3C*. The molecular characterization of these genes in patients with a phenotype resembling CSS and overlapping with its clinical features will vindicate their clinical associations. Eventually, to unveil the biological functions of these genes and to delineate their contributions to the various clinical phenotypes will require their targeted disruption, singly and in combination, in animal models.

## Supporting Information

Figure S1 **Effect of rs2110277 (A/G) on transcription factors binding sites in the** ***C7orf58*** **alternative promoter.** The PROMO computer prediction software shows that the A and G alleles differ in their binding of the transcription factors early growth response 1–3 (EGR), glucocorticoid receptor beta (GR-β), yin-yang (YY1) activator/repressor, CCAAT-enhancer-binding proteins (CEBPα, CEBPβ).
(TIF)

Table S1 **Amplicon sizes and DNA sequences of PCR primers used for the amplification and sequencing of exons, surrounding introns, untranslated sequences and immediate promoter regions of the** ***WNT16*** **,** ***FAM3C*** **and** ***C7orf58*** **genes.** The locations of the primers within each gene are donated by their closest exon (Ex), promoter (P) or untranslated (UTR) regions.
(PDF)
