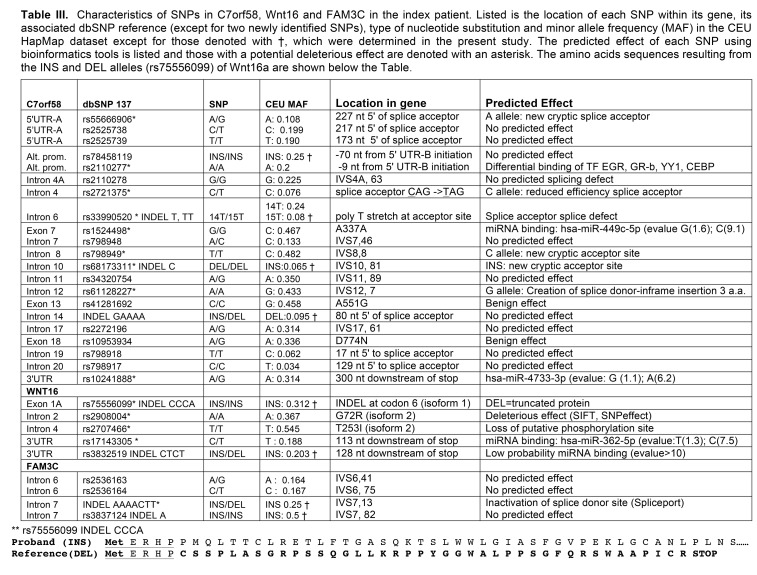# Correction: Duplication of *C7orf58*, *WNT16* and *FAM3C* in an Obese Female with a t(7;22)(q32.1;q11.2) Chromosomal Translocation and Clinical Features Resembling Coffin-Siris Syndrome

**DOI:** 10.1371/annotation/c8463645-79f3-4d9b-bc55-d5d53e43a9ba

**Published:** 2013-09-04

**Authors:** Jun Zhu, Jun Qiu, Gregg Magrane, Malak Abedalthagafi, Andrea Zanko, Mahin Golabi, Farid F. Chehab

A Table and it's associated legend were omitted from the article. They are available here: 

**Figure:**