# Supplementary figures and images for: Duplication of C7orf58, WNT16 and FAM3C in an Obese Female with a t(7;22)(q32.1;q11.2) Chromosomal Translocation and Clinical Features Resembling Coffin-Siris Syndrome
Source: PLoS One. 2012 Dec 27;7(12):e52353. doi: 10.1371/journal.pone.0052353 (PMC3531478; doi:10.1371/journal.pone.0052353)

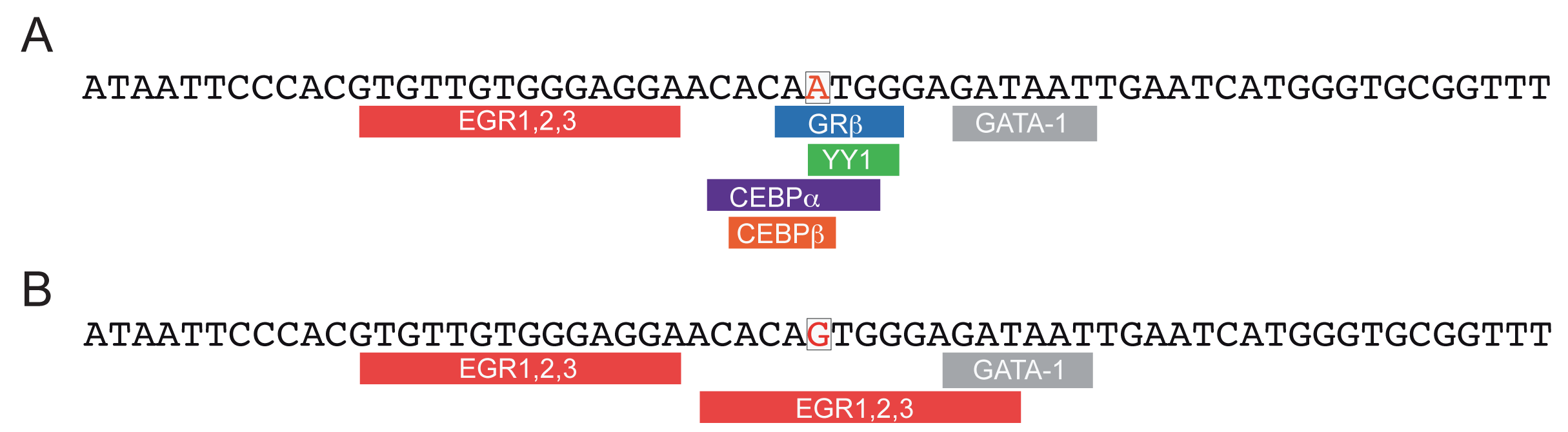

Supplement: Figure S1 — Effect of rs2110277 (A/G) on transcription factors binding sites in the C7orf58 alternative promoter. The PROMO computer prediction software shows that the A and G alleles differ in their binding of the transcription factors early growth response 1–3 (EGR), glucocorticoid receptor beta (GR-β), yin-yang (YY1) activator/repressor, CCAAT-enhancer-binding proteins (CEBPα, CEBPβ). (TIF) [file pone.0052353.s001.tif]
