# Supplementary material for: Duplication of C7orf58, WNT16 and FAM3C in an Obese Female with a t(7;22)(q32.1;q11.2) Chromosomal Translocation and Clinical Features Resembling Coffin-Siris Syndrome
Source: PLoS One. 2012 Dec 27;7(12):e52353. doi: 10.1371/journal.pone.0052353 (PMC3531478; doi:10.1371/journal.pone.0052353)
Supplement: Table S1 — Amplicon sizes and DNA sequences of PCR primers used for the amplification and sequencing of exons, surrounding introns, untranslated sequences and immediate promoter regions of the WNT16 , FAM3C and C7orf58 genes. The locations of the primers within each gene are donated by their closest exon (Ex), promoter (P) or untranslated (UTR) regions. (PDF) [file pone.0052353.s002.pdf]

	Forward primer	Reverse primer	Size (bp)
WNT16			
Ex 1A	ACGATCATCACCTGCTGACA	CCTGATCAAATCCCCAAATC	461
Ex 1-2	AGGGATAACTTGGTGCTGGAC	GAACCGGTGGAGGAGTTGT	688
Ex 3	GAACAAGAGCAAATCTTCCTTTC	ACTTGAGGGCTGCCAGTGT	567
Ex 4	TCAAGAGGAAATAGACCCAGAAA	AAGGCTGGATGGAGTGGTTA	590
Ex 4, 3'UTR	ACAAGGCAGAGAATGCAACC	TCAACATCTTCTATCCACACACAA	698
FAM3C			
5'UTR	CGAGCTGGCTTTCTCCTG	GGAGGCTGAAGTCGTAGGTG	265
Ex 1	ATATGCCCCATTTCAAACTCAA	CCTAAAAACAAAGGGCTGAATG	279
Ex 2	AAACTAGGCAGATGGTTCAAGC	CACATTTCGTTTTCTAGCACTGA	454
Ex 3	TTGAACACTTATTTTTGTGGACCT	GCAATTTCTAAAGCAGTTTTTCC	238
Ex 4	GCTCTTTGTGGTTTGAGATTTG	TTTCAAAATATCCATTAAACTCAGG	295
Ex 5	GCAGCACTGAACAATTGAAAAT	AGGGCTTTATCATCAAGAGCAG	
Ex 6	TGAATTCACATCTTCTCTGCTAATG	TGAACTAACGCAAATAGCATCA	274
Ex 7	TGAGATTTGTCTGTCTTTTGGA	CAGGAAGGAGGCAAAGTAAAAA	383
Ex 8	TCTCATTTTGTCTCATGTTTTGTG	GGATGGGAAAATGTACCGTAAG	296
Ex 9	TTTGAAGGTCTGCCATGTATGT	TACTCATGCACACACCAAGATG	392
3'UTR	AGCTGTAAACCAAGTTGGAAGC	TGCTTGAAAAACCTTGTGTGAG	400
C7orf58			
P1	AAAAGGCAGTGCCATGAATC	TCGGTTGGGAGAAATGAAAG	342
P2	TTGCATCCACCAAAACGATA	CAGTGGGCTTTGTCCAGAAT	667
P3	CCCTGGGTATCCAGAGTTCA	GCTAGCATTTCCCCAAATGA	613
P4	CCTCATTTGGGGAAATGCTA	TTGCTGATGAAAACCACTCG	308
P5	CGAGTGGTTTTCATCAGCAA	CAGCATTGAAGAACGCAAAA	362
P6	CAACAGGCACTTTGGGTTTT	TTTCCCCAACTCCAATCAAG	372
Ex 1	TTGGAGTTGGGGAAAAAGAA	CATCAGGTCACTCTCTAGGCTGT	499
Ex 2	CCAAATAGCCCTTGATTTGC	TCTATGATGTGAGAAAGTTGGTCAT	409
Ex 3	TTGAGATCATTTACTTGTGTTTCAGA	TGGCCAGTTGGAAGATTCTC	362
Ex 3A	CTCCCAGGTTCAAGCAATTC	TTGCACATTTTTGCCTGAAG	506
Ex 4A alt	ATGCTGTTTGTTCCCTCCAG	CTGCTGCTGAAGACATACGC	545
Ex 4	AAGCTACTTCATTATTCTGGATTTG	TGAACTGCGGAAGATCTGTG	389
Ex 5	GGCAAACAAAGGCCTCTCTAA	GAAAGTCAATGATGGCATTCTTTAT	486
Ex 6	GCAACTGAAATTGTTCAAGGAA	TGCTTCCAGTTCATTTTTAAACC	500
Ex 7	CAAGACATTTAAACCTTTCTCTTTCA	TGGCCACATATGTTTAGAAGATCA	493
Ex 8	CTGCATAGTATTCCATGGTGTG	TTAATTGTCTGAACCACAGTGAA	392
Ex 9	AAAACTGTTGACTTCCAAAAAGA	TCTTAGTCCAGAGGTCAGAAAAA	470

Ex 10	TATCCAGGTCCTCCCTGTCA	GCATATAAATCTTGCTCATTTTGC	489
Ex 11	GGCATTTACATCAACAACTTTGC	AAAGACATTTATTTTCCTGAGCA	481
Ex 12	TGCCAGTTTTGAGGATTGAA	TCTCTCCCTTAGGTCGTTAG	401
Ex 13	GTTTACAGTTTGACTTAGAAGTGTTC	TGCAAAAGTGATAGATACTGACTCC	369
Ex 14	TGCAGCCACAAATCAACTATG	AGGGGAAAGTTCACATCCTG	484
Ex 15	ACCCTGGCAGGTGGTTAATA	TACCTGGCTGGGATTCTCAC	588
Ex 16	GCTAGCAGGGGAAAGATAAGG	TGTCAATAAAAAGTAATGCCCTTG	374
Ex 17	CTATTTGGTTGGCAGGAAGCC	CGGGAATTGTTCAGGCTATT	484
Ex 18 alt	GGTATTTGGCCATGAGACTGA	TTGGAATAGGTCAAACCCAAT	287
Ex 19	GGAACAGGTTTGGGAGAGAA	TGCACTGCCTCACATTTCAC	589
Ex 20	GCAGTCGTCACTGCTCAACT	AACAACCATGTTGTGACTGGA	485
Ex 21	GTCTTGTATCCAGTCACAACATGG	AATTGGTGGGGTAAGGGAAC	494
Ex 22	CATCTTTATTTAAGAAATGCATGTGA A	CCTTGGCTGAATGATGATGA	452
Ex 23	GCAACATCTGTGTGGCAGAG	TACTGGTGTGCATGCATGTGCT	461
3'UTR-A	TGATGGCTTAGGCTTTGTGA	CAAATGCTTGGTGGGAAAAA	452
3'UTR-B	ATAGGTGCTTTTTCCCACCA	ATGGAGAGGCTGCACACATT	706
3'UTR-C	AGCTAGTCACCCGGACAATG	CCCTTGTGGAACATTCCTGT	545
